# Pure Air a Medicine

**Published:** 1861-09

**Authors:** 


### PURE AIR A MEDICINE.

ON one occasion an English family became ill in mid-winter. Medical advice was obtained, and the usual remedies applied for a long time, without producing any marked favorable change. All the physicians who heard of the circumstances were greatly puzzled to explain the case satisfactorily, even to themselves. At length, a pane of glass was accidentally broken in the only room of the house, and the inmates were so much taken up with their troubles, that it was either not noticed or there was not time or disposition or ability to repair the damage. All at once, however, the sick began to improve; the doctor's eyes were simultaneously opened a little wider, and he gave orders to let the window alone, with the result that in a short time every member was entirely well.

Let every invalid who is as "'fraid as death" of a puff of pure air, bear this suggestive incident in wise remembrance, the balance of his days; or if an open door or window is not practicable, at least keep open the fire-place, and either have a little fire in it, or a liberal lamp or a brisk jet of gas burning in it; this causes a draft up the chimney, and is a safe, easy, and efficient way of ventilating any sick-room; a ventilation which would save valuable lives, in multitudes of instances.

